# Aquaporin-1 regulates platelet procoagulant membrane dynamics and in vivo thrombosis

**DOI:** 10.1172/jci.insight.99062

**Published:** 2018-05-17

**Authors:** Ejaife O. Agbani, Christopher M. Williams, Yong Li, Marion T.J. van den Bosch, Samantha F. Moore, Adele Mauroux, Lorna Hodgson, Alan S. Verkman, Ingeborg Hers, Alastair W. Poole

**Affiliations:** 1 School of Physiology, Pharmacology and Neuroscience, University of Bristol, Biomedical Sciences Building, Bristol, United Kingdom.; 2 Department of Physiology and Pharmacology, Cumming School of Medicine, University of Calgary, Calgary, Alberta, Canada.; 3 Wolfson Bioimaging Facility, University of Bristol, Biomedical Sciences Building, Bristol, United Kingdom.; 4 Departments of Medicine and Physiology, University of California San Francisco, San Francisco, California, USA.

**Keywords:** Cell Biology, Hematology, Coagulation, Platelets, Thrombosis

## Abstract

In response to collagen stimulation, platelets use a coordinated system of fluid entry to undergo membrane ballooning, procoagulant spreading, and microvesiculation. We hypothesized that water entry was mediated by the water channel aquaporin-1 (AQP1) and aimed to determine its role in the platelet procoagulant response and thrombosis. We established that human and mouse platelets express AQP1 and localize to internal tubular membrane structures. However, deletion of AQP1 had minimal effects on collagen-induced platelet granule secretion, aggregation, or membrane ballooning. Conversely, procoagulant spreading, microvesiculation, phosphatidylserine exposure, and clot formation time were significantly diminished. Furthermore, in vivo thrombus formation after FeCl_3_ injury to carotid arteries was also markedly suppressed in AQP1-null mice, but hemostasis after tail bleeding remained normal. The mechanism involves an AQP1-mediated rapid membrane stretching during procoagulant spreading but not ballooning, leading to calcium entry through mechanosensitive cation channels and a full procoagulant response. We conclude that AQP1 is a major regulator of the platelet procoagulant response, able to modulate coagulation after injury or pathologic stimuli without affecting other platelet functional responses or normal hemostasis. Clinically effective AQP1 inhibitors may therefore represent a novel class of antiprocoagulant antithrombotics.

## Abstract 2

AQP1 controls platelet procoagulant response and modulates coagulation after injury or pathologic stimuli without affecting other platelet functional responses or normal hemostasis.

## Introduction

Central to the role of platelets in hemostasis and thrombosis is the dramatic morphological transformation they undergo upon contact with subendothelial collagen to generate balloon-like as well as procoagulant-spread structures (1–6). Ballooning increases the surface area for phosphatidylserine (PS) exposure, and together with procoagulant-spread structures, it enhances microvesiculation and amplifies thrombin generation by the provision of extensive procoagulant surfaces for binding of the prothrombinase complex. Computer models of changes in cell shape based on cortical tension, water, and ion entry have shown that these deformations are pressure driven and can be predominantly mediated by water entry (7). Accordingly, we showed that the mechanism underlying the morphological procoagulant transformations of activated platelets is driven by a coordinated system of Na^+^, Cl^–^, and water entry (4), and the molecular drivers of salt entry are likely calcium-activated chloride channels and nonspecific cation channels (4). The molecular pathway for water entry required for procoagulant membrane remodeling is not clear, but water channel aquaporins are the most likely candidates. Of these, aquaporin-1 (AQP1) is a family member with significant levels of transcript expression and detection by proteomics in platelets and megakaryocytes (8–10).

The aquaporins (AQPs) are a family of passive integral membrane proteins that, in many species, facilitate the highly efficient yet strictly selective passage of water and small solutes across cell membranes (11, 12). There are 13 mammalian AQPs, including the aquaglyceroporins, which are a subset that facilitate the passive transport of glycerol and small solutes, such as urea and carbon dioxide (12, 13). The first of the AQPs discovered was human AQP1, and it is of the subfamily of pure water channels (14, 15). Like other AQPs, it forms tetramers to function as a water pore. The aromatic/arginine selectivity filter is the narrowest part of this pore; it is only 2.8-Å wide but large enough for the passage of a single water molecule (16). The structure and function of AQPs have been reviewed extensively (16, 17). The distribution of AQPs in mammalian tissue is broad, with notable expression in red blood cells and the vessel wall (13). Data from AQP-knockout mice and from humans with loss-of-function mutations in AQPs demonstrate their role in epithelial fluid secretion, cell migration, and edema. This suggests that modulation of AQP expression or function may have broad clinical indications, such as in the treatment of cancer, obesity, brain injury, glaucoma, and intracranial hypertension (13). There is some evidence of selectivity in AQP function; for example, while human and mouse AQP1 regulate cell volume and membrane dynamics in a variety of cell types (18), there is no evidence that AQP4 regulates cell volume. Notably, there are no useful AQP1 inhibitors, and although several putative small-molecule inhibitors have been reported (19, 20), these compounds lack selectivity and specificity and are largely cytotoxic (13, 21, 22). Therefore, convincing evidence for the role of individual AQPs in platelet procoagulant membrane dynamics and thrombosis will require the use of gene knockout models (4).

In this study, we utilized AQP1-knockout mice generated by targeted gene disruption (23) and revealed through dynamic high-resolution imaging that AQP1 deletion diminished procoagulant spreading, microvesiculation, and in vivo thrombus formation, while having minimal effects on platelet secretion, aggregation, and normal hemostasis. AQP1 mediated these events through its facilitation in collagen-stimulated platelets of rapid membrane swelling/stretching after initial salt entry. This, in turn, promoted calcium entry via mechanosensitive cation channels TRPC6 and Piezo1, which is required to achieve the sustained cytosolic calcium required for the full platelet procoagulant response. We conclude that the water channel AQP1 is a suitable target for the control of thrombotic events and, that when available, clinically safe and effective AQP1 inhibitors may represent a novel class of antiprocoagulant antithrombotics for the management of arterial thrombotic disease, including acute coronary syndrome, coronary artery disease, and stroke.

## Results

### The expression and subcellular localization of AQP1 in platelets.

**Figure 1:**
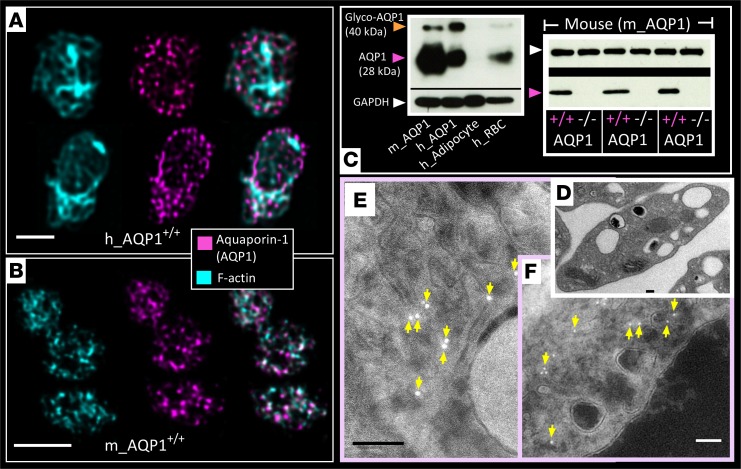
Expression of the water channel aquaporin-1 in human and mouse platelets. (**A** and **B**) Representative images of unstimulated (**A**) human and (**B**) mouse platelets probed for F-actin (phalloidin; cyan blue) and aquaporin-1 (AQP1) (magenta) by confocal immunofluorescence microscopy. Two and three platelets are shown in **A** and **B**, respectively. (**C**) Representative immunoblot of human and mouse platelet lysates for AQP1 (at 28 kDa) and glycosylated AQP1 (at 40 kDa), with GAPDH as loading control. m_AQP1, mouse AQP1; h_AQP1, human AQP1. (**C**) Human adipocyte and human red blood cell lysates were used as negative and positive controls, respectively. Blots show human (h_AQP1) and C57Bl/6 mouse platelet AQP1 (m_AQP1). Immunoblots for AQP1 in mouse platelets show absence of expression in AQP1^–/–^ mice. Blots are representative of 3 independent experiments. (**D**) Transmission electron microscopy of platelets isolated from WT mice is shown. (**E** and **F**) Wild-type mouse platelets processed by the Tokuyasu method for immunogold labeling. AQP1 located on the membrane structures of the open canalicular structure indicated by yellow arrows in reverse contrast images. Data were from platelets obtained from 6 (**A** and **B**) or 3 independent experiments (**C–F**). Scale bar: 3 μm (**A** and **B**); 100 nm in (**D–F**).

Data on the presence of AQP1 in platelets are sparse and limited in scope by the lack of molecular evidence (24, 25). Here, we demonstrate the expression of AQP1 in mouse and human platelets by immunocytochemistry using confocal immunofluorescence microscopy (Figure 1, A and B) and stimulated emission depletion (STED) microscopy (Supplemental Figure 1, A and B; supplemental material available online with this article; https://doi.org/10.1172/jci.insight.99062DS1). The immunofluorescent signal was absent in platelets isolated from AQP1-null mice (Supplemental Figure 1A), validating the specificity of the antibody. Expression of AQP1 in human and mouse platelets and its absence in AQP1-null mouse platelets was further confirmed by Western blot (Figure 1C). We next sought to establish the subcellular localization of AQP1 by immunogold staining transmission electron microscopy. Figure 1, D–F, shows platelet AQP1 localized to a membrane network, likely to be the open canalicular system (OCS).

### Deletion of AQP1 did not diminish normal platelet secretion or aggregation.

**Figure 2:**
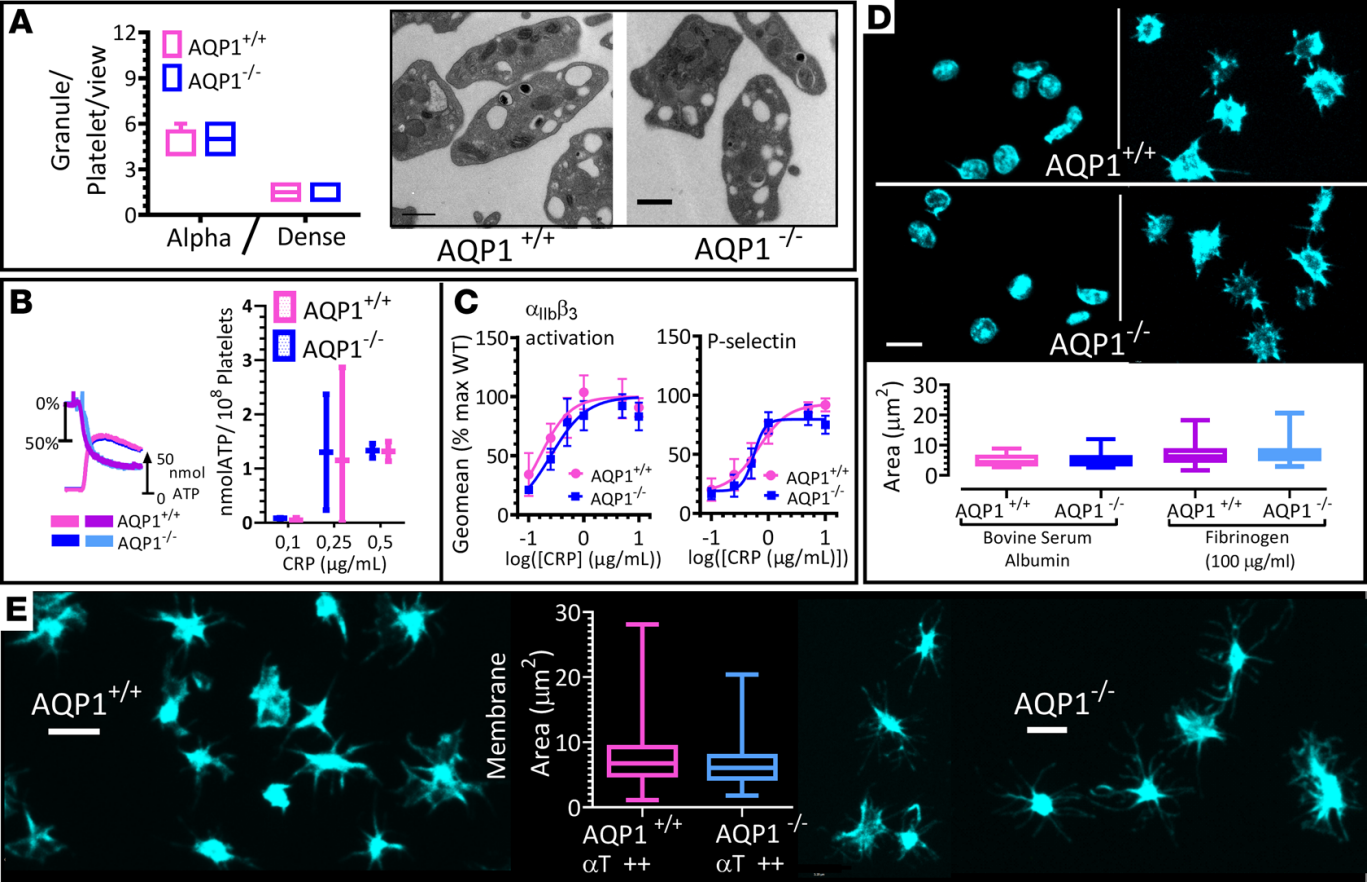
Ablation of AQP1 does not alter integrin activation or secretion in mouse platelets. (**A**) Transmission electron microscopy of AQP1^+/+^ and AQP1^–/–^ mouse platelets. Representative images are shown, and granule count was determined. (**B**) Ablation of AQP1 had no effect on platelet-dense granule release. AQP1^+/+^ and AQP1**^–/–^** mouse platelets were stimulated with 0.5 μg/ml collagen-related peptide (CRP), and ATP secretion was assessed by luminometry. Representative trace and graph with levels of secretion (mean ± SEM). Blue and cyan tracings of chart show percentage aggregation and the simultaneous ATP release recorded in AQP1^+/+^ mouse platelets, respectively. Corresponding tracings for AQP1**^–/–^** platelets are shown in light and deep magenta. (**C**) Washed mouse platelets (5 × 10^7^/ml) from AQP1^+/+^ or AQP1^–/–^ mice were stimulated for 10 minutes with a range of concentrations of CRP in the presence of 1 mM CaCl_2_. Integrin α_IIb_β_3_ activation and P selectin exposure were measured by flow cytometry. The geometric mean of the fluorescence intensity was determined, and data are shown as the percentage of maximal control (AQP^+/+^) response. Curves were fitted by F test. (**D**) Washed platelets from wild-type (AQP1^+/+^) or AQP1-null (AQP1^–/–^) mice allowed to adhere to BSA-coated (2%) or fibrinogen-coated (100 μg/ml) surfaces and stained for actin (FITC-phalloidin). Quantification of spreading (surface area) in box-and-whisker plots. (**E**) Platelets adherent to BSA were stimulated with 1 U/ml thrombin and spreading analyzed as in **D**. Statistical significance was determined by 2-way ANOVA and Bonferroni post hoc test (**C**) and by Wilcoxon signed-rank test (**A**, **B**, and **E**). Scale bar: 500 nm (**A**); 3 μm (**D** and **E**). Data were from 6 independent experiments.

Water channels have been reported to mediate the vesicular swelling that regulates content expulsion during secretion in several secretory cells (26, 27). There is evidence for the involvement of AQP7 in a human platelet secretion defect (28), but there has not yet been a report of how AQP1 may modulate platelet secretion or aggregation. Constitutive deletion in mouse of AQP1 did not affect hematological parameters, such as white or red blood cell count or mean platelet or red cell volume; nor did it affect platelet levels of key surface glycoproteins (Supplemental Figure 2). We then confirmed by electron microscopy that AQP1-null platelets showed a similar distribution and number of α and dense granules as wild-type controls (Figure 2A). Similarly, granular release after platelet activation via glycoprotein VI remained normal (Figure 2, B and C). Under these conditions, integrin α_IIb_β_3_ activation and platelet aggregation remained unchanged in AQP1^–/–^ platelets by comparison with wild-type platelets, demonstrating that integrin inside-out signaling was intact (Figure 2C). Next, we studied platelet spreading on a fibrinogen-coated surface to investigate integrin outside-in signaling (Figure 2, D and E) but did not observe a significant effect of AQP1 deletion. Together, these data indicate that AQP1 is not involved in the regulation of these platelet functions. This finding is consistent with our proposed role for AQP1 primarily as a mediator of the procoagulant membrane response, rather than platelet secretion or aggregation.

### Procoagulant spreading and microvesiculation, but not ballooning, is attenuated by the deletion of AQP1.

**Figure 3:**
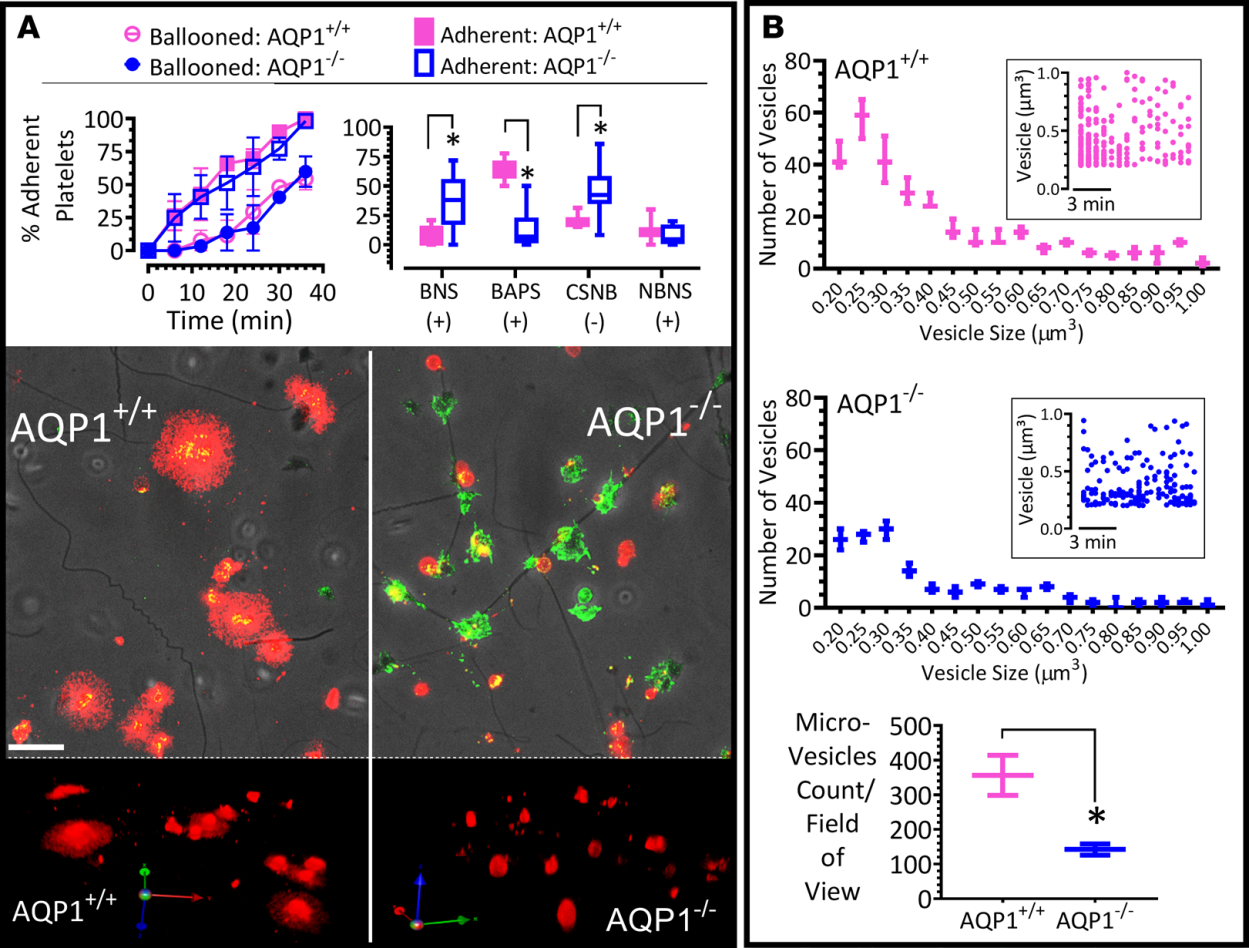
Platelet procoagulant spreading and phosphatidylserine exposure but not membrane ballooning is markedly diminished by AQP1 ablation. (**A**) Time course of membrane ballooning in platelets adherent to collagen in Tyrode medium containing 1 mM calcium, with 4 platelet phenotypes identified as previously reported (4–6). BNS, ballooned nonspread platelets; BAPS, ballooned and procoagulant-spread platelet; CSNB, conventional-spread nonballooned platelet; NBNS, nonballooned nonspread platelets. +, annexin V^+^ status; -, annexin V^–^ status. Images show a typical field of view of AQP1^+/+^ and AQP1^–/–^ mouse platelets adherent to collagen at the 1-hour time point, with fluorescent annexin V (red) superimposed with corresponding P selectin images (green) and phase-contrast images. 3D images showing annexin V staining. Images were obtained by spinning-disk confocal microscopy (original magnification, ×100). (**B**) AQP1^+/+^ and AQP1^–/–^ platelets adhering to a collagen-coated surface were monitored for microvesicle release in real time by imaging-based particle counting; at the 1-hour time point, microvesicles were identified (over a 70 × 90 μm field of view of extended focus images), as annexin V^+^ particles with diameter between 100 nm and 1 μm. Data are displayed as histogram and inset scatter plots. Box-and-whisker plot shows a comparison of microvesicles identified from adherent AQP1^+/+^ and AQP1^–/–^ platelets. Data analysis was performed by Wilcoxon signed-rank test. *P < 0.05 was considered significant. Scale bar: 10 μm. Data were from 7 independent experiments.

Studies of different cell types reveal a role for AQP1 in cell migration in vitro and in vivo (29). For example, targeted disruption of AQP1 reduced migration of aortic endothelial cells and limited tumor angiogenesis and growth (30, 31). The underlying mechanism involved AQP support for osmotic water flow across the plasma membrane in cell protrusions that form during migration and in the formation of filopodia and lamellipodia (29, 32). Given that our data demonstrate the localization of AQP1 in platelets to be primarily on membranes structures (Figure 1D), we investigated whether AQP1 was involved in the actin remodeling and membrane dynamics of the platelet procoagulant response, which amplifies coagulation (4) and may be dysregulated in the absence of AQP1. To address this, we utilized high-resolution 4D live-cell imaging in tandem with pharmacological or physiological manipulations to monitor in real time the procoagulant membrane dynamics of platelets as they adhere to fibrillar collagen. Data from AQP1^+/+^ and AQP1^–/–^ mouse platelets showed that ablation of AQP1 did not diminish the rate or extent of procoagulant membrane ballooning (Figure 3A). However, platelet procoagulant spreading and microvesiculation were significantly reduced (Figure 3). Water entry is therefore an integral part of the mechanism for the platelet procoagulant membrane dynamics, as we had previously reported (4).

### Deletion of AQP1 results in reduced thrombus formation after carotid artery injury.

**Figure 4:**
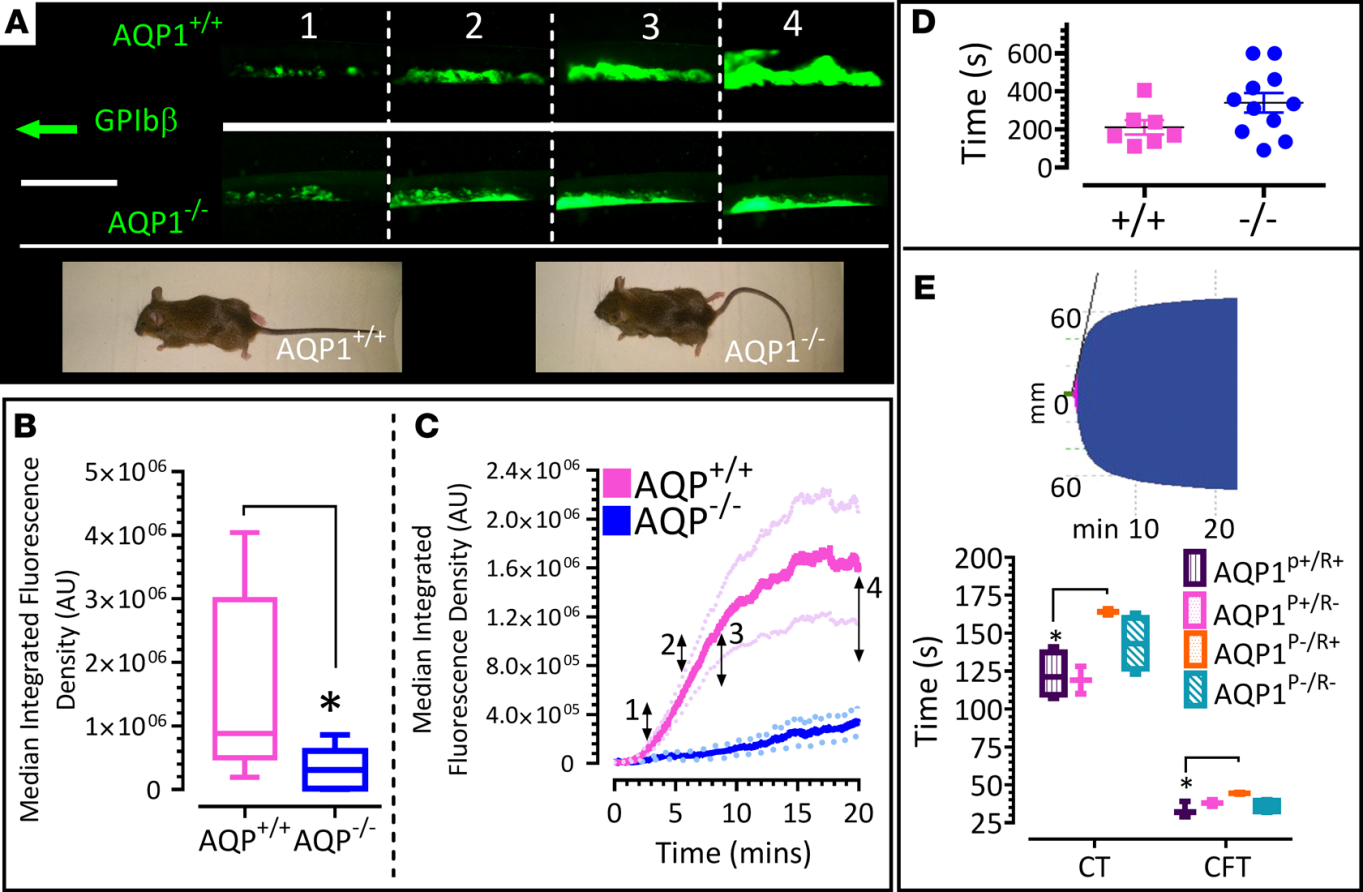
Constitutive (whole animal) ablation of AQP1 suppresses thrombus formation in vivo and in vitro without affecting hemostasis. (**A–C**) Mice were administered DyLight 488–conjugated anti-GPIbβ antibody to label platelets. Carotid artery damage was achieved by treatment with FeCl_3_. Fluorescently labeled platelets were imaged by intravital microscopy. Images at frames indicated in **A** correspond to time points indicated in **C**. Wild-type (AQP1^+/+^) and AQP1-null (AQP1^–/–^) mice images show comparable growth rates and gross morphology. (**B**) Median fluorescence integrated density (MFID) is shown as interleaved box-and-whisker plots, with whiskers showing minimum to maximum values, medians, and interquartile ranges. (**C**) The time course of change in MFID (median and minimum and maximum values) for thrombus formation in AQP1^+/+^ and AQP1^–/–^ mice. (**D**) Tail bleed times were assessed, and data shown are mean ± SEM of time to stop bleeding. (**E**) Ablation of AQP1 in platelets delayed clotting times in intrinsic pathway analyses of whole mouse blood coagulation. The inset shows thromboelastometry (ROTEM) data; the histogram shows clotting time (CT) and clot formation time (CFT) for freshly drawn, citrated whole AQP1^+/+^ and AQP1^–/–^ mouse blood. Using a platelet-rich plasma (PRP) swap approach, blood is reconstituted as shown. AQP1^P+/R+^ indicates AQP1^+/+^ PRP combined with AQP1^+/+^ red and other cells (RBC); AQP1^P+/R–^ indicates AQP1^+/+^ PRP combined with AQP1^–/–^ RBC; AQP1^P–/R+^ indicates AQP1^–/–^ PRP combined with AQP1^+/+^ RBC; and AQP1^P–/R–^ indicates AQP1^–/–^ PRP combined with AQP1^–/–^ RBC. Data analysis was performed by Wilcoxon signed-rank test. Scale bar: 2 mm. *P < 0.05 was considered significant. Data were from 8 independent experiments.

Platelets are a central cell in the generation of arterial thrombi; however, it was important to determine whether the diminished procoagulant response associated with deletion of AQP1 would affect in vivo thrombosis. Using constitutive AQP1^–/–^ and littermate-matched AQP1^+/+^ mice, we assessed thrombus formation in vivo by intravital microscopy of carotid arteries after ferric chloride injury and analyzed rate of thrombus formation, thrombus fragility, and embolization. Figure 4 shows a substantial suppression (by 79.3% ± 8.8%) of thrombus formation in vivo in the absence of the AQP1 gene (Figure 4, A–C). Tail bleed times, however, showed no difference after injury, indicating normal hemostatic function (Figure 4D). Tail bleed times are dependent upon several hemostatic mechanisms, in addition to the procoagulant platelet response, including arterial and arteriolar constriction. It is possible, therefore, that the tail bleed assay misses critical features that rely upon a procoagulant response; however, with this caveat, there is no effect of AQP1 gene deletion on a physiological hemostatic response. In vitro platelet exchange experiments showed a platelet-specific delay in clotting times and clot formation times in blood samples lacking AQP1 gene (Figure 4E). These data suggest platelet-expressed AQP1 modulates thrombosis after injury or pathologic stimuli without affecting normal hemostasis. We were therefore keen to elucidate the mechanism by which this water channel may selectively regulate platelet-driven thrombosis.

### AQP1 expression mediates faster platelet swelling kinetics and enhanced cytosolic calcium responses after hypotonic stimulus.

**Figure 5:**
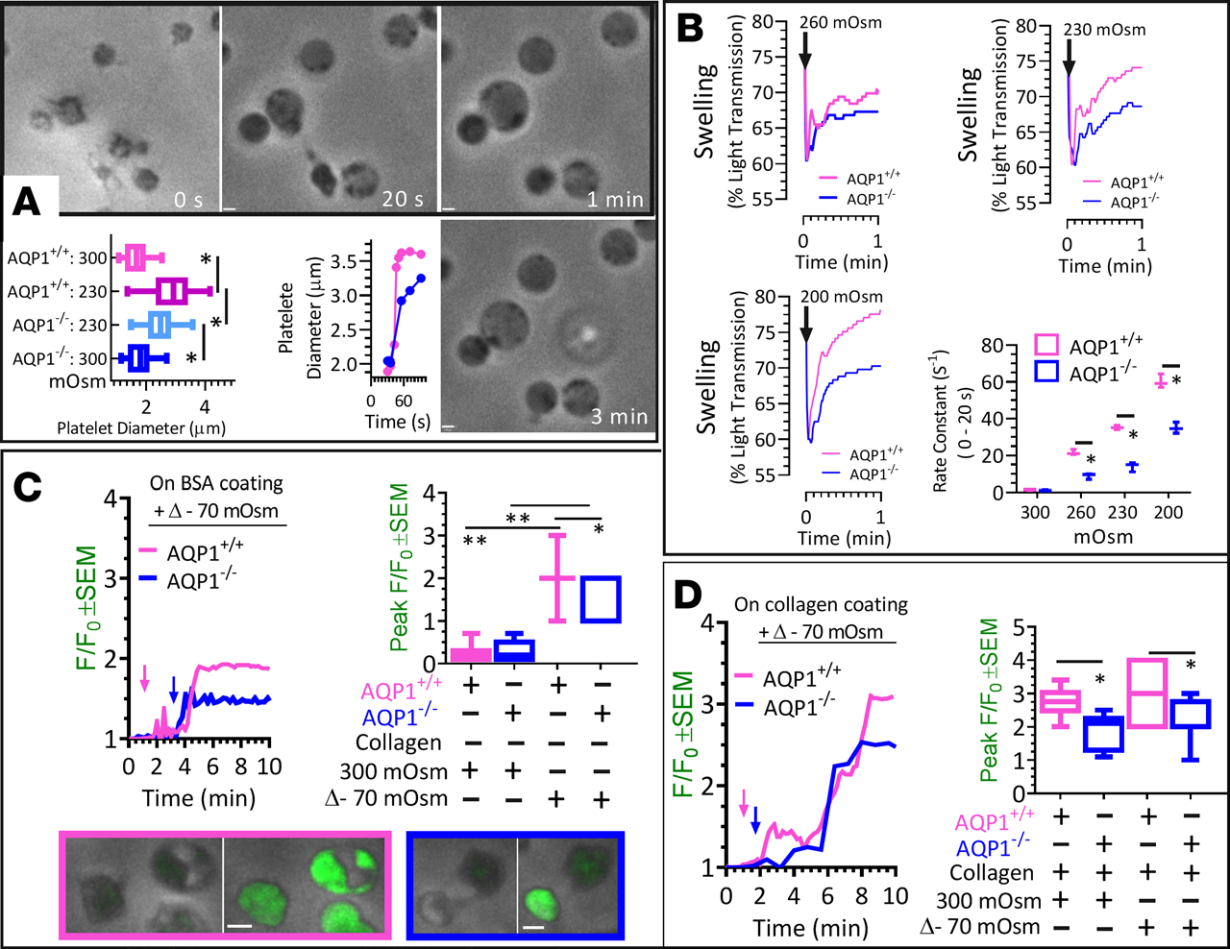
Platelet aquaporin-1 mediates rapid swelling kinetics after hypotonic shock. (**A**) Platelets adherent to serum-coated dishes were challenged with a Δ-70 mOsm osmotic change, cell swelling was monitored by phase-contrast microscopy, and images at indicated time points are shown. The box-and-whisker plot shows AQP1^+/+^ and AQP1^–/–^ platelet diameter after 3 minutes of hypotonic shock. The diameter versus time graph shows the time course of change in diameter (blue symbols indicate AQP1^–/–^ platelets; magenta symbols indicate AQP1^+/+^ platelets). (**B**) Using an optical aggregometer, light transmittance was determined in platelet suspensions in modified Tyrode solutions. Baseline light transmittance was recorded before and after AQP1^+/+^ and AQP1^–/–^ platelet suspensions were exposed to hypotonic shock (Δ-40, Δ-70 and Δ-100-mOsm osmotic change). Percentage light transmission values were recorded over time and plotted as shown in representative graphs. The first-order rate constants of these plots were derived for the first 20 seconds after hypotonic shock and plotted as shown in the box-and-whisker plot. (**C**) Time courses of cytosolic calcium signals in untreated platelets adherent to bovine serum-coated surfaces and exposed to normotonic or a 70-mOsm osmotic gradient stimulus; here, normotonic and hypotonic Tyrode were supplemented with 1 mM calcium. Arrows in the F/F_0_ graph indicate time points for induction of hypotonic stimulus. In addition, the box-and-whisker plot of peak calcium mobilized in platelets is shown. From left to right, representative images of AQP1^+/+^ and AQP1^–/–^ platelets at basal and at peak cytosolic calcium signal are shown. (**D**) As in **C**, but platelets were monitored as they adhered to collagen fibers. The box-and-whisker plot shows peak calcium mobilized in platelets. Arrows in time-course graph indicate the time point of platelet adherence to collagen fiber. Data analysis was performed by Wilcoxon signed-rank test. *P < 0.05, ** P < 0.01 were considered significant. Scale bar: 1 μm (**A** and **C**). Data were from 4 independent experiments.

The speed of cell swelling as well as the magnitude of membrane stretch or shrinkage after tonic challenges can be markedly altered by the expression of the AQPs (33–35). Here, we investigated whether the expression of AQP1 facilitated osmoregulation in platelets, consistent with the function of water channels in other cell types (34, 35). Using washed platelets from AQP1-null mice and litter-matched wild-type controls, we assessed the rate of water influx after hypotonic stimulus and determined platelet swelling by microscopy and light transmission assays. The results shown in Figure 5, A and B, revealed that the rate and extent of platelet swelling was markedly higher in platelets expressing AQP1. Alone, a 2-minute exposure of platelets to 70-mOsm osmotic gradient increased AQP1^+/+^ and AQP1^–/–^ platelet diameter; however, the extent of swelling in AQP1^+/+^ platelets was greater compared with platelets lacking AQP1 (Figure 5A). Similarly, the first-order rate constants of platelet swelling determined by optical aggregometer were markedly higher in the presence of AQP1 after a 40-mOsm osmotic gradient challenge (20.4 ± 2.7 S^–1^ vs. 9.6 ± 1.3 S^–1^). Similar elevations in the rate of swelling was recorded with 70-mOsm (34.0 ± 6.1 S^–1^ vs. 15.2 ± 4.4 S^–1^) and 100-mOsm (57.0 ± 2.5 S^–1^ vs. 32.1 ± 1.9 S^–1^) osmotic gradients (Figure 5B). These results indicate a role for AQP1 in platelet swelling and shape change after osmotic challenges (Figure 5B). In parallel experiments, we monitored calcium mobilization after hypotonic stimulus in platelets settled on a BSA-coated surface (Figure 5C) and in platelets adhering to collagen in hypotonic Tyrode supplemented with 1 mM CaCl_2_ (Figure 5D). Alone, a hypotonic stimulus increased the peak calcium response in both human (data not shown) and mouse platelets (Figure 5C) and AQP1 expression significantly increased calcium mobilization in these platelets whether in contact with BSA- or collagen-coated surfaces (Figure 5, C and D). Together, these data indicate a role for AQP1 not only in facilitating shape change and osmoregulation in platelets but also in enhancing the calcium signaling required for procoagulant membrane dynamics.

### AQP1 is localized proximal to mechanosensitive cation channels, and its deletion or mechanosensitive cation channel blockade attenuated extracellular calcium entry and PS exposure.

**Figure 6:**
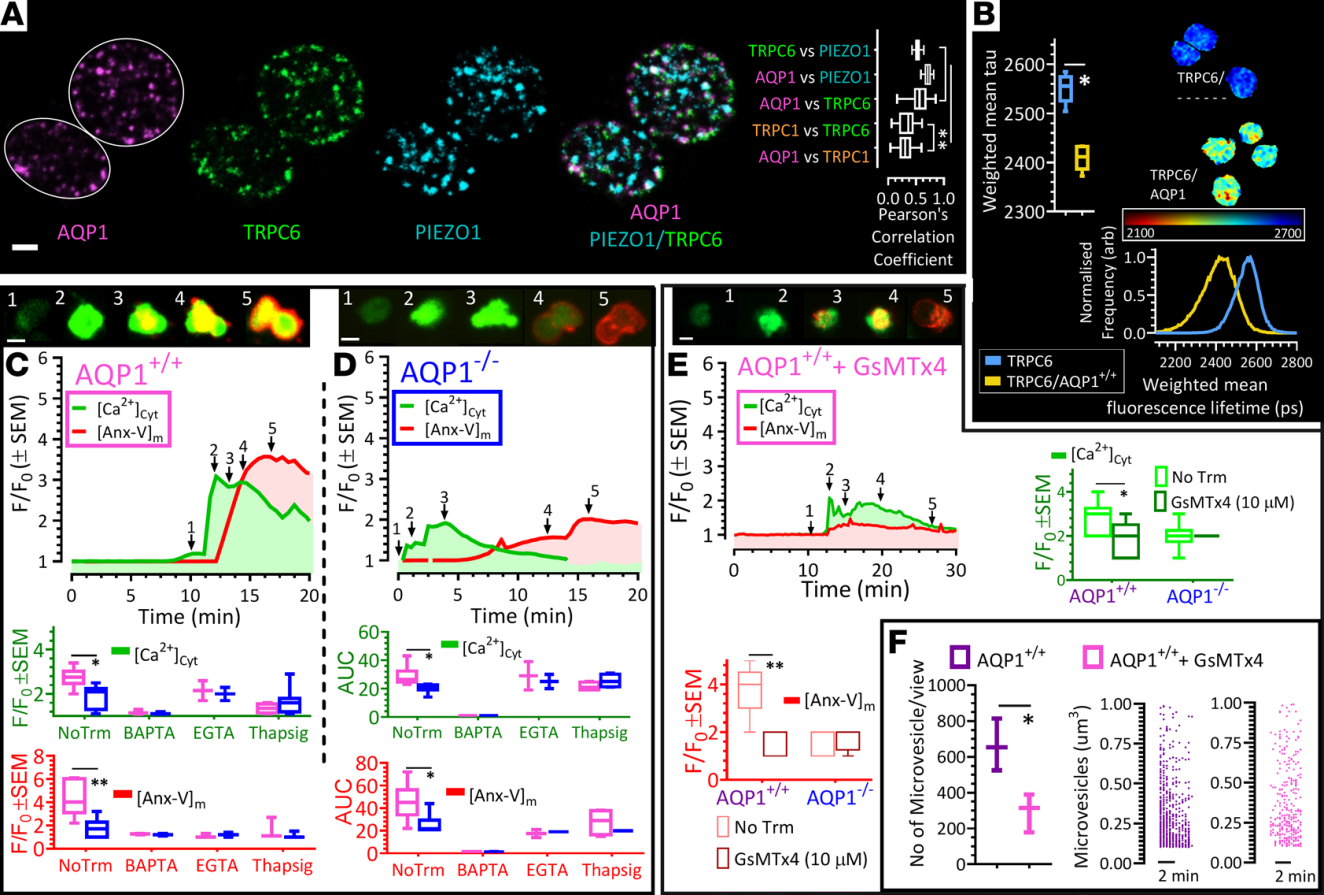
AQP1 is localized in proximity to mechanosensitive cation channels, and its deletion or mechanosensitive cation channel blockade attenuates extracellular calcium entry, PS exposure, and microvesiculation. (**A**) Human platelets were stained with antibodies against AQP1, TRPC6, and Piezo1, and images were evaluated for colocalization. Pearson’s correlation analysis demonstrates AQP1 to be in proximity to TRPC6 and Piezo1 but not in significant proximity to TRPC1. (**B**) AQP1 interaction with TRPC6 in human platelets after evaluation by Förster resonance energy transfer (FRET). (**C** and **D**) Washed mouse platelets in modified Tyrode supplemented with 1 mM calcium were labeled with the calcium indicator Fluo-4 (green) and Alexa Fluor 568–annexin V (AnxV, red) and allowed to adhere to collagen-coated surfaces. (**C** and **D**) Platelets superimposed for AnxV and Fluo-4 at time points after adherence to collagen corresponding to the numbered arrows in the graphs below. Data are for AQP1^+/+^ (magenta) and AQP1^–/–^ (blue) platelets. Box-and-whisker plots of peak change and area under the curve (AUC__15 min_) of calcium responses in untreated platelets (NoTrm) and in platelets pretreated with the cytosolic calcium chelator BAPTA-AM (1 μM), extracellular calcium chelator EGTA (1 mM), and sarco/endoplasmic reticulum Ca^2+^ ATPase (SERCA) inhibitor thapsigargin (1 μM) in AQP1^+/+^ and AQP1^–/–^ platelets. Similarly, the peak change and AUC of annexin V binding were quantified and shown. (**E**) Mouse platelets were preincubated with the mechanosensitive channel inhibitor Grammostola spatulata mechanotoxin 4 (GsMTx-4) or vehicle as a control; outcome is shown as in **C** and **D**. (**F**) Microvesiculation was quantified in control (untreated) and GsMTx4-treated AQP1^+/+^ mouse platelets. Data analysis was performed by Wilcoxon signed-rank test. *P < 0.05, **P < 0.01 were considered significant. Scale bar: 1 μm (**A** and **B**). Data were from 4 independent experiments.

The mediation of intracellular calcium entry by AQPs has been reported in astrocytes (33) and in a subset of retinal glial cells (35); both reports implicated mechanosensitive cation (MSC) channels as part of a molecular complex that integrated osmoregulation and calcium signaling (33, 35). Here, we examined the possibility that a similar mechanism exists in platelets. To begin with we aimed to visualize transient receptor potential C1 (TRPC1) and C6 (TRPC6) and piezo1 in human and mouse platelets (Figure 6A). From our immunocytochemistry data, we determined, as previously reported (36), the percentage of signal colocalization of AQP1 versus TRPC6 and AQP1 versus piezo1; the results were comparable to but significantly higher than similar measures of AQP1 and TRPC1 colocalization (Figure 6A). We then studied the possibility of molecular interactions between AQP1 and MSC channel proteins by Förster resonance energy transfer/fluorescence lifetime imaging (FRET/FLIM), which estimates intermolecular interactions within ≤10 nm. The results summarized in Figure 6B showed that the fluorescent lifetime of TRPC6 was significantly reduced in the presence of AQP1. We obtained similar results in our probe for an interaction between the mechanosensitive channels AQP1 and Piezo1 in platelets (data not shown). These data suggest the likelihood of an interaction between AQP1 and the MSC channels TRPC6 and piezo1, although it remains unknown from these experiments whether AQP1 directly interacts with MSC channels or is part of a molecular complex for the regulation of calcium entry in platelets.

Next, we examined the spatiotemporal changes in cytosolic calcium of washed platelets from AQP1^+/+^ and AQP1^–/–^ mice, as they adhered to fibrillar collagen in Tyrode of physiological tonicity, supplemented with 1 mM calcium. As previously reported (4, 5), collagen increased cytosolic calcium in these platelets (Figure 6, C and D), due to both store release and calcium entry, since chelation of intracellular calcium by BAPTA or platelet preincubation with the noncompetitive inhibitor of the sarco/endoplasmic reticulum Ca^2+^ ATPase (SERCA) thapsigargin markedly reduced the calcium response (Figure 6, C and D). Notably, when compared with wild-type controls, calcium mobilization was significantly attenuated in the absence of AQP1 (Figure 6, C and D) but not to the same degree as that observed with BAPTA (Figure 6, C and D). This suggested that Ca^2+^ release, attenuated by preincubation with thapsigargin, remained viable even after AQP1 deletion. Furthermore, the peak and the AUC of the calcium response were comparable between AQP1^+/+^ and AQP1^–/–^ platelets once extracellular calcium was chelated with 1 mM EGTA. This indicated that extracellular calcium entry, but not store calcium release, were markedly reduced by AQP1 deletion (Figure 6, C and D). Given our previous observation that the platelet procoagulant membrane remodeling is preceded by a sustained rise in cytosolic calcium (4), the present data are consistent with a role for AQP1 in facilitating calcium entry.

In these experiments, we simultaneously monitored platelet spatiotemporal exposure of PS by recording the real-time accumulation of annexin V on platelet membranes, along with changes in cytosolic calcium. Figure 6, C and D shows a delay as well as significant reductions (AQP1^+/+^ vs. AQP1^–/–^: 4.3 ± 0.6 vs. 1.7 ± 0.3) in membrane annexin V accumulation in the absence of AQP1. Our data are thus consistent with previous reports demonstrating the requirement of a sustained elevation in cytosolic calcium for a full procoagulant response in collagen-stimulated platelets (2, 37). Furthermore, in agreement with a recent report on the role of MSC channels in platelet calcium signaling (38), we hypothesized that calcium entry through the MSC channels TRPC6 and piezo1 may be a major contributor to the sustained elevated calcium required for the full procoagulant response. Similar to effects of platelet AQP1 ablation on calcium mobilization and PS exposure, we recorded in collagen-stimulated AQP1^+/+^ platelets preincubated with the pan-MSC channel inhibitor GsMTx4 (39), reductions in the amplitude and AUC of intracellular calcium mobilized over time (Figure 6E), the concomitant accumulation of annexin V (Figure 6E), and microvesiculation (Figure 6F). The effects of AQP1 deletion on the platelet procoagulant response are therefore phenocopied by blockade of MSC channel activity.

## Discussion

The ability of platelets to support thrombin generation and localize coagulation to wound sites is hinged on the rapid and dramatic shape change they undergo to form ballooned membranes, procoagulant-spread structures, and microvesicles. Together, these are the extended surface area that externalize PS and become an assembly site for the binding of coagulation factors and the amplification of coagulation (2, 4–6, 40). In this study, we demonstrate an important role for the water channel AQP1 in this procoagulant remodeling. First, we showed that human and mouse platelets express AQP1 in a manner that indicates their localization to internal membranes, likely to be the OCS. Second, our data reveal that deletion of AQP1 does not affect platelet functions that are targets for current antiplatelet therapies in stroke and cardiovascular disease (CVD) management. In this regard, mice lacking AQP1 showed normal hemostatic responses after blood vessel injury, and the platelets from these mice showed no defects in aggregation or secretion from α or dense granules. Third, our data demonstrate that, although procoagulant membrane ballooning remains unaffected by AQP1 deletion, full platelet procoagulant response after pathological injury was suppressed. Thrombus formation after ferric chloride injury to carotid arteries was massively reduced in vivo; in addition, the ability of platelets to procoagulant spread and microvesiculate after collagen stimulation was attenuated in the absence of AQP1. Thus, data from this study demonstrate a specific role for AQP1 in the membrane-swelling events that control procoagulant spreading, but not ballooning.

The present study reveals that AQP1 accelerated the agonist-induced platelet membrane swelling and enhanced the calcium entry required for procoagulant cytoskeletal remodeling. Our EM data suggest that AQP1 is not expressed on the plasma membrane but rather on the OCS, and superresolution images appear to confirm this by showing AQP1 as connected punctae (Supplemental Figure 1). Although expressed on the OCS, AQP1 can elicit the functional consequences reported in this study, because the intricate internal membrane system of the OCS is contiguous with the plasma membrane and essentially functions as an extended plasma membrane. Platelet OCS forms multiple connections with sites on the platelet surface (41) and represents a membrane reservoir that can be evaginated onto the platelet surface (42) but also a fusion site for secretory granules and potentially a site for expression of functional membrane channels. In this way, AQP1 channels expressed on the OCS membrane system are already in position for water conductance.

We have previously characterized the dynamics of membrane ballooning (4), and we estimated that the rate of membrane ballooning is unlikely to be limited by water channels only, given the narrow pore size and rate of water passage through these channels (13, 17, 43). In the platelet procoagulant response, it is more likely that water entry is initially through water channels; however, as ballooning progresses, water also permeates through the lipid bilayer. AQP1 and the isoform AQP7, which we also found to be expressed in platelets (Supplemental Figure 3), are likely to be involved in this early phase of water entry. Our data suggest a redundant role for AQP1 in procoagulant membrane ballooning, as this event proceeded unabated after AQP1 deletion. A double or triple AQP-knockout model would be required to establish the role of AQP in this early phase of procoagulant membrane ballooning. Furthermore, unlike membrane ballooning and conventional spreading events, which were unaffected by AQP1 deletion (Figures 2 and 3), PS exposure, procoagulant spreading, and the subsequent microvesiculation were tightly regulated by AQP1 alone (Figures 3 and 4). This suggests some specificity in the role of different AQP isoforms, which are often coexpressed with each other in different cell types (13). Perhaps the spatial distribution of these isoforms may explain their differential function.

**Figure 7:**
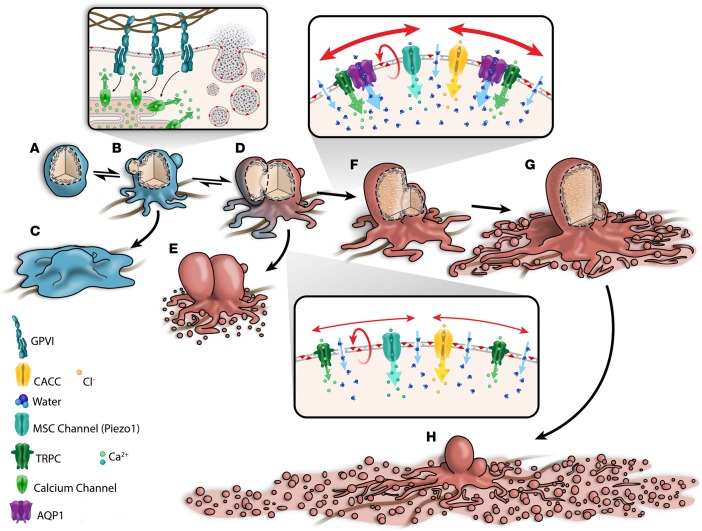
Water entry via aquaporin-1 mediates a stretch-induced amplification of calcium entry and a full procoagulant response in collagen-activated adherent platelets. Upon contact with subendothelial collagen, platelets (**A**) signal via glycoprotein receptor VI to cause a rise in cytosolic calcium and the activation of nonspecific cation channels as well as Ca^2+^-activated chloride channels (CACC), resulting in an initial salt entry, which is then followed by the influx of water along its concentration gradient. Water channel AQP1 facilitates rapid water entry and cell swelling, leading to stretching of the plasma membrane, which then activates the opening of mechanosensitive cation (MSC) channels. Of these, TRPC6 of the family of transient receptor potential cation (TRPC) channels and Piezo1 channels are likely candidates (39) that open to allow additional influx of extracellular calcium, sustaining the rise in cytosolic calcium (**B–D**), which is critical for activation of the lipid “scramblase,” leading to PS externalization (6) (**D** and **E** and **D–H**). The internal hydrostatic pressure initiates membrane swelling at regions of high calpain activity (6, 56, 57). In combination with external osmotic pressure, this leads to full-scale irreversible membrane ballooning (**D–G** and **D** and **E**). Ballooning is temporally correlated with the formation of the expansive procoagulant surface, which subsequently breaks up as a result of multiple coalescence events to form PS^+ve^ microvesicles (4, 5, 40). (**F–H**) Both ballooned nonspread (BNS; **F**) and ballooned procoagulant-spread platelets (BAPS; **H**) increase the procoagulant area mediating the acceleration of coagulation at wound sites (4, 5, 40). Data from this study indicate that AQP1 has a specific role in the membrane-swelling events that control procoagulant spreading but not ballooning. Upon ablation of AQP1, platelet swelling kinetics are slowed and unable to or only weakly activate MSC channels for enhanced Ca^2+^ entry. This results in limited Ca^2+^ influx and procoagulant spreading, thus favoring the formation of the BNS platelet phenotype (**D** and **E**) and limited thrombosis.

Earlier, we reported on the pivotal role of calcium in the platelet procoagulant response of collagen-stimulated platelets (4, 5). In this study, glycoprotein VI–induced cytosolic Ca^2+^ rise was sufficient to drive membrane ballooning in wild-type and AQP1-null platelets; however, the formation of procoagulant-spread structures and microvesiculation was severely limited in platelets lacking AQP1 (Figure 3). Under physiological conditions, it is likely that the full complement of water channels is required to facilitate calcium entry and a full procoagulant response, which consists of membrane ballooning, procoagulant spreading, and microvesiculation (4–6), and a lack of AQP1 limits the extent of this remodeling (Figure 7). A rise in Ca^2+^ in response to osmotic swelling is a well-reported observation in mammalian cells (44). AQP1 induced rapid swelling and a greater amplitude of calcium influx, which could be inhibited by GsMTx4 blockade of MSC channels (Figures 5 and 6). Given that AQP1 is impermeable to ions, other channels must therefore mediate this swelling-induced Ca^2+^ entry. We speculate that the specific action of AQP1 in the platelet procoagulant response is mediated via calcium-permeable stretch-activated channels, several of which are expressed in platelets (39, 45–47). MSC channels have been demonstrated to bind to and functionally interact with several isoforms of AQPs (33, 35, 48). Here, we confirmed the colocalization and fluorescence resonance energy transfer activity of the AQP1 and MSC channels TRPC6 and piezo1 (Figure 6A), which suggest the likelihood of their functioning in concert to regulate platelet response to osmotic changes and calcium entry. It is not clear how other molecular players translate the differential AQP1-induced calcium signaling to regulate procoagulant response. One possibility is a direct interaction of AQP1 with members of the transmembrane protein (TMEM) family, which may act as calcium-dependent lipid scramblases or calcium-activated chloride channels, either of which will limit procoagulant response in events of attenuated Ca^2+^ entry (49, 50). Our initial investigations had revealed a direct interaction between AQP1 and TMEM40 (data not shown); however, this gene remains uncharacterized, and its molecular function is unknown. Further work beyond the scope of the current study is needed to unravel the function of this TMEM and the nature of its interaction with AQP1.

A finding of major clinical significance is the secretion-sparing action of AQP1 deletion while thrombus formation is massively suppressed. Over 25% of patients on antiplatelet drugs go on to suffer an ischemic event (51). In addition, a major clinical side effect associated with current antithrombotic regimens in the management of CVD is the significant bleeding resulting from the use of dual antiplatelet therapy, for example, aspirin and P2Y_12_ blockers usually targeting the inhibition of platelet secretion (52). This status quo demonstrates a need for alternative targets for the control of thrombotic events associated with CVD. Furthermore, platelets secrete a plethora of releasates essential for the maintenance of vascular integrity and cardiovascular hemostasis (53). Consequently, new antithrombotic approaches that spare essential platelet secretion may attain greater clinical effectiveness and limit or eliminate the major side effects of current antiplatelet therapies. While there is some evidence for the involvement of AQP-6 in human platelet secretion (28), the current data suggest that AQP1 may be a novel alternative antithrombotic target, since its deletion essentially spared platelets secretory functions and yet suppressed pathological thrombus formation in the same model. There are still no real AQP1 inhibitors (13, 19, 20); presently, only mercurial compounds target AQP1, but these are cytotoxic (13, 20). Work in this area in ongoing; thus, once identified, clinically safe and effective AQP1 inhibitors may represent a novel class of antiplatelet drugs for the management of acute coronary syndrome, coronary artery disease, and stroke.

### Conclusions.

AQP1 is a major regulator of the platelet procoagulant membrane dynamics and the prothrombotic response. Suitable AQP1 inhibitors may therefore represent a novel class of antiprocoagulant antithrombotics.

## Methods

### Materials.

Collagen Reagens HORM Suspension was from Takeda. Cross-linked collagen-related peptide was synthesized by Richard Farndale (University of Cambridge, Cambridge, United Kingdom). FITC–P selectin and PE-JON/A antibodies for flow cytometry were from Emfret Analytics. Alexa Fluor 568–conjugated annexin V and Fluo-4 AM were purchased from Life Technologies. Glass bottom 35-mm dishes (P35G-1.5-14-C) were obtained from MatTek Corporation. Rabbit polyclonal (IgG1) anti-AQP1 was from Alomone Lab Ltd. (catalog AQP-001) and Santa Cruz (H-55, catalog sc-20810). Ezrin/radixin/moesin (ERM), phospho-ERM^T567/T564/T558/48G2)^ (catalog 3726) and phospho-PKC substrate (catalog 38938) antibodies were from Cell Signaling Technologies (New England Biolabs). Far-red/infrared secondary antibodies (donkey anti-mouse, catalog 715-625-150; donkey anti-human, catalog 709-625-149) were from Jackson Immunoresearch. The following Alexa Fluor secondary antibodies were from Thermo Fisher Scientific: Alexa Fluor 488 (goat anti-rabbit, catalog A-11008; goat anti-mouse, catalog A-11001; donkey anti-rabbit, catalog A-21206; donkey anti-goat, catalog A-11055; donkey anti-mouse, catalog A-21202), Alexa Fluor 568 (goat anti-rabbit, catalog A-11036; donkey anti-rabbit, catalog A10042; donkey anti-sheep, catalog A-21099; goat anti-mouse, catalog A-21134), and Alexa Fluor 647 (goat anti-mouse, catalog A-21235; goat anti-rabbit, catalog A27040; donkey anti-mouse, catalog A-31571; goat anti-rabbit, catalog A-21245; chicken anti-rabbit, catalog A-21443; chicken anti-goat, catalog A-21469). Chronolume (P/N 395) was from Chrono-log Corporation (Labmedics). NuPAGE SDS/PAGE sample buffer (catalog LA0041) was from Invitrogen. Pieizo1, TRPC1, and TRPC6 blocker, GsMTx4 (catalog 4912), was from Tocris. All other reagents were from Millipore unless otherwise indicated

### Human platelet-rich plasma and washed platelet preparation.

Blood was drawn into 4% trisodium citrate (1:9, v/v) and washed platelets were prepared as previously described (4, 5). Platelets were resuspended at 4 × 10^8^/ml in modified HEPES-Tyrode buffer (135 mM NaCl, 3 mM KCl, 10 mM HEPES, 5 mM glucose, and 1 mM MgCl_2_.6 H_2_O, pH 7.3) containing 10 μmol/l indomethacin, 0.02 U/ml apyrase, and 0.1% (w/v) D-glucose.

### Animals.

AQP1-knockout mice were generated by targeted gene disruption, as previously reported (23), and were provided for this study by Alan S. Verkman (University of California San Francisco). Genotype analysis of ear-punch DNA was done by PCR at weaning age. Wild-type littermates (AQP1^+/+^) were used as controls. Wild-type and AQP1-null (AQP1^–/–^) mice of mixed gender were used for experimentation at age 8–10 weeks.

### Mouse washed platelet preparation.

Mice (8–10 weeks, 20–25 g) were sacrificed by rising CO_2_ inhalation, in accordance with Schedule 1 of the Animals (Scientific Procedures) Act (1986), and blood was drawn from the inferior vena cava into 4% trisodium citrate (1:9, v/v). Prior to platelet preparation, full blood counts were conducted using a Pentra ES60 hematology analyser (Horiba Medical), and values were adjusted for anticoagulant volume. Washed platelets were prepared as previously described (4). Platelets were resuspended at 4 × 10^8^/ml in modified HEPES-Tyrode buffer (135 mM NaCl, 3 mM KCl, 10 mM HEPES, 5 mM glucose, and 1 mM MgCl_2_.6 H_2_O, pH 7.3) containing 10 μmol/l indomethacin, 0.02 U/ml apyrase, and 0.1% (w/v) D-glucose.

### Lumi-aggregometry.

Aggregation and ATP secretion were measured simultaneously using a Chrono-log (490-4D) aggregometer and luciferin/luciferase reagent (Chronolume) at 37°C under stirring conditions. Washed platelets (2 × 10^8^/ml) were preincubated with inhibitors or vehicle control (0.1% DMSO) for 10 minutes at 37°C, prior to agonist stimulation. ATP measurements were calibrated using a 2 nmol ATP standard.

### Measurement of platelet swelling and shape change.

Platelets adherent on serum-coated dishes were challenged with a 70-mOsm osmotic gradient, and cell swelling and shape change was monitored by phase-contrast microscopy. Images were calibrated and platelet diameters were derived with the use of the line tool in Volocity 6.3 software. In parallel experiments using an optical aggregometer (Chrono-log Corporation), platelet swelling in response to hypotonic shock was indirectly estimated by the changes in light transmission of platelet suspension in modified Tyrode (300 mOsm). Briefly, 250 μl platelet suspension at 300 mOsm was placed in a cuvette maintained at 37°C; baseline light transmittance was then recorded for 20 seconds, after which platelets were exposed to a hypotonic shock of 260, 230, and 200 mOsm by the addition of an equal volume of Tyrode solution of appropriately lowered NaCl concentrations. Percentage light transmission values were recorded over time and plotted, and from these first-order rate constants were derived. Mean platelet volume was also determined using a Pentra ES60 hematology analyser (Horiba Medical).

### Glutathione-S-transferase pull-down and proteomic assays.

Human AQP1 glutathione-*S*-transferase (AQP1-GST) recombinant protein (20 μg, LifeSpan BioSciences Inc.) or GST alone was incubated with 0.5 ml of Glutathione Sepharose 4B (GE Healthcare) in RIPA buffer (25 Mm HEPES, pH 7.4, 0.2 m NaCl, 2.5 mM MgCl_2_, 1% NP-40, 0.5% sodium deoxycholate, 10% glycerol, 1 mM PMSF, 25 mM sodium fluoride, 1 μM microcystin-LR, and complete protease inhibitor cocktail) at 4°C overnight with gentle top-over-bottom rotation. The sepharose beads were spun down at 500 *g* for 5 minutes at 4°C and washed 3 times with 1 ml RIPA buffer, and 30 μl AQP1-GST or GST sepharose beads alone were incubated with 250 μl human platelets lysates (4 × 10^8^ platelets/ml) in 0.5 ml RIPA buffer at 4°C overnight with gentle rotation. The sepharose beads were then sedimentated and washed as described above and subject to tandem mass tag proteomic or immunoblotting analysis.

### Protein extraction and immunoblotting.

Washed platelets (4 × 10^8^/ml) stimulated as indicated were lysed in 4×NuPAGE sample buffer containing 0.5 M DTT. Lysates were analyzed by SDS-PAGE/Western blotting using Bis-Tris gels as previously described (54) and visualized by near-infrared detection (Odyssey CLx, LI-COR). For quantification of immunoblotting, densitometry was performed using LI-COR Image Studio 5 Software.

### Immunocytochemistry.

Platelet suspension at 1 × 10^7^ was dispensed onto MatTek dishes or coverslips precoated with BSA or agonist as indicated and allowed to adhere for 2 hours at 37°C in CO_2_ incubator. Excess platelets suspension was aspirated, and adherent platelets were fixed with 2% paraformaldehyde at room temperature for 10 minutes. This was followed by gentle rinses 3 times with wash buffer (modified Tyrode containing 0.01% saponin). Platelets were then permeabilized by a 5-minute exposure to 0.1% saponin in modified Tyrode and gently rinsed 3 times with wash buffer. Blocking of nonspecific binding was achieved by the application of block buffer (2% BSA in wash buffer) for 2 hours at room temperature. This was followed by the application of primary antibody and overnight incubation at 4°C. Platelets were thereafter rinsed gently 3 times with wash buffer and incubated with suitable secondary antibody for 1 hour at room temperature. Finally, coverslips or dishes were rinsed 3 times, mounted with curing agent (ProLong Diamond), and stored protected from light at 4°C until viewed.

### Flow cytometry.

Integrin α_IIb_β_3_ activation and α-granule secretion were measured using a PE-conjugated antibody (JON/A) directed against the high-affinity form of integrin αIIbβ3 and a FITC-conjugated antibody (Wug.E9) for the α-granule marker CD62P (P selectin). Washed mouse platelets (1 × 10^6^/ml in modified HEPES-Tyrode buffer containing 10 μmol/l indomethacin, 0.02 U/ml apyrase, 0.1% [w/v] D-glucose, and 1 mM CaCl_2_) were stimulated with agonist (10 minutes) in the presence of FITC-JON/A and PE-CD62P before fixation with 1% paraformaldehyde for 30 minutes. Two-color fluorescent analysis was conducted on a FACSCalibur flow cytometer (BD Biosciences) using FACSDiva software. Based on forward and side scatter, the platelet population was gated and 20,000 events were captured.

### Live platelet imaging.

Platelets were preincubated (10 minutes) with probes as indicated in figures and legends. Fibrillar collagen (20 μg/ml) was used to precoat MatTek dishes and then blocked with 2% fatty acid–free BSA. Aliquots of washed platelets suspended in Tyrode supplemented with 1 mmol/l CaCl_2_ were added onto dishes, and platelet activities were monitored in *xy* or *xyz* dimensions over time. High-resolution 3D and 4D images of live-platelets adhering over agonist-coated surfaces were obtained at 25°C using spinning-disk confocal system as previously described (4, 5).

### Image deconvolution and analysis.

Image resolution was improved by the restoration complement of Volocity 6.3 imaging software (Perkin-Elmer). Point-spread functions specific to the microscope, refractive index of immersion medium, and acquisition lens numerical aperture were calculated using the action menu of the software; this was then used to conduct iterative restoration of the fluorescent images. Using preinstalled Volocity algorithms, platelet-derived microvesicles were identified by intensity in 4D data using both Fluo-4 and annexin V signal intensities, combined with a size discrimination step for vesicles sized between 100 nm and 1 μm, which excluded vesicles <100 nm and >1 μm from the analysis output.

### STED microscopy.

Imaging was performed on a Leica STED 3× system (Leica Microsystems). Excitation was provided by a white-light laser with a repetition rate of 80 MHz, and an acousto-optical beam splitter selected excitation wavelengths of 488 nm and 561 nm for excitation of Alexa Fluor 488 and Alexa Fluor 568, respectively. Images were acquired using a 100× 1.4 NA oil immersion objective. Fluorescence was detected using time-gated hybrid detectors over an emission range of 492–550 nm for Alexa Fluor 488 detection and 568–650 nm for Alexa Fluor 568 detection. Notch filters centered on 488 nm and 568 nm minimized any laser scatter. STED depletion was achieved through use of 592 nm (for Alexa Fluor 488) and 660 nm (for Alexa Fluor 568) depletion lasers.

### FRET/FLIM.

FLIM was used to measure changes in FRET, enabling the discrimination of different states/environments of ERM proteins in relation to AQP1 proteins. Fluorescence lifetime images were acquired on a Leica TCS SP8 system attached to a Leica DMi8 inverted microscope (Leica Microsystems). Excitation was provided by a white-light laser, with a repetition rate of 20 MHz, and an acousto-optical beam splitter selected an excitation wavelength of 488 nm. Images were acquired using a 63× 1.4 NA oil immersion objective. Fluorescence of Alexa Fluor 488 was detected using a hybrid detector operating in photon-counting mode over an emission range of 495–530 nm. A notch filter centered on 488 nm minimized any laser scatter into the detector. Time resolved data were acquired through use of a PicoHarp 300 TCSPC module (PicoQuant) controlled through SymPhoTime64 software (PicoQuant). FLIM images were acquired with 256 × 256 pixels and 4,096 time bins. Fitting of FLIM images was performed with the FLIMfit software tool developed at Imperial College London (55). Temporal binning of the fluorescence decays was performed prior to fitting, resulting in 256 time bins per decay. Global analysis fitting of the images was then performed image wise, with a double exponential model on all pixels above an intensity threshold of 100 photons, allowing spatial variations in the intensity-weighted mean fluorescence lifetime to be visualized.

### Sample processing for embedding in EPOXY resin.

Platelet-rich plasma was collected and spun at 590 *g* for 5 minutes. Supernatant was removed from the platelet pellet, and the pellet was fixed in 2.5% glutaraldehyde in 0.1 M phosphate buffer (PB) (pH7.4). The pellet was washed in PB and then incubated in 1% osmium tetroxide in PB for 30 minutes. After washing in PB and deionized water, the pellet was incubated in 3% uranyl acetate in deionized water for 30 minutes. After washing with deionized water, the pellet was dehydrated in a graded series of increasing amounts of ethanol (70%, 80%, 90%, 96%, 100%, and 100%, with each step lasting for 10 minutes). After removal of the 100% ethanol, the pellet was incubated with pure Epon for 2 hours at room temperature. Thereafter, the Epon was replaced with fresh Epon, and this was hardened overnight in a 60°C oven. Subcellular morphology of platelets in ultrathin counterstained sections were imaged with a Tecnai Spirit T12 microscope (FEI) and analyzed with Fiji software. Granule numbers were quantified manually and expressed as granules/cell/image.

### Immunogold labeling of Tokuyasu cryosection.

Platelet pellets, formed by spinning platelet-rich plasma at 590 *g* for 5 minutes were fixed with 2% paraformaldehyde, 0.2 glutaraldehyde in 0.1 M PB for 1 hour at room temperature. Cell pellets were washed with PBS before incubation in 0.15% glycine/PBS for 10 minutes and thereafter resuspended in warm 12% gelatin at 37°C for 10 minutes before centrifugation to reform pellets. Cell pellets were subsequently cooled on ice for 30 minutes, and 0.5-mm gelatin blocks were cut using a razor blade. Blocks were infused with 2.3 M sucrose at 4°C for 16 hours before mounting onto aluminium pins and freezing in liquid nitrogen. Ultrathin cryosections were cut using a EM UC6 microtome equipped with a FC6 Cryo unit (Leica Microsystems). Sections were picked up in a 1% methylcellulose/1.75 M sucrose solution and mounted onto carbon-coated pioloform films on copper mesh grids. Grids were incubated on a drop of PBS at 37°C for 30 minutes. Grids were transferred between drops of 0.1% glycine/PBS and 1% BSA/PBS for 10 and 30 minutes, respectively. Sections were labeled with AQP1 antibody diluted in 0.1% BSA/PBS for 1 hour at room temperature before washing in 0.1% BSA/PBS. Grids were subsequently incubated in 10 nm Protein A Gold (Cell Microscopy Centre) diluted in 0.1%BSA/PBS for 20 minutes. Grids were washed in 0.1% BSA/PBS and distilled water to remove unbound gold before counterstaining in 0.3% uranyl acetate in 1.8% methylcellulose on ice for 5 minutes. Grids were finally air dried using the wire loop method.

### Ferric chloride carotid injury model in mouse.

Mice were anesthetized with ketamine 100 mg/kg (Vetalar V, Pfizer) and 10 mg/kg xylazine (Rompun, Bayer). Platelets were labeled by intravenous administration of 100 mg/kg Dylight-488–conjugated anti-GPIbβ antibody, 10 minutes prior to induction of thrombosis. Right carotid arteries were exposed, and 2 × 1 mm 12% ferric chloride-soaked filter paper was placed on the arterial adventitia for 3 minutes. Time-lapse microscopy of the injury site for 20 minutes was performed, and images were processed using ImageJ (NIH). Background fluorescence values measured upstream of the injury site were subtracted from thrombus-specific fluorescence, and data were expressed as integrated densities. Median integrated fluorescence density was determined as the thrombus area multiplied by the fluorescence intensity values above background for that area.

### Tail bleeding.

Experiments were conducted on 25–35 g male and female mice. Mice were anesthetized (100 mg/kg ketamine and 10 mg/kg xylazine intraperitoneally), and a transverse incision was made with a scalpel 5 mm from the tip of the tail. The tail was immersed in normal saline (37°C) in a hand-held test tube, and the time from incision to cessation of bleeding was recorded, up to a maximum of 10 minutes.

### Statistics.

Data were analyzed using GraphPad Prism 7 and are presented as box-and-whisker plots showing minimum to maximum values, medians, and interquartile ranges of data. Normality was assessed by the D’Agostino and Pearson omnibus as well as the Shapiro-Wilk tests. We determined statistical significance by the Friedman test, followed by the Dunn multiple comparison test or Wilcoxon signed-rank test. *P* values of less than 0.05 were considered significant.

### Study approval.

Human blood was obtained from healthy drug-free volunteers, who gave full informed consent in accordance with the Declaration of Helsinki, with approval from the local research ethics committee of the University of Bristol. Mice were bred and maintained in the University of Bristol animal facility in accordance with United Kingdom Home Office regulations. All procedures were undertaken with United Kingdom Home Office approval in accordance with the Animals (Scientific Procedures) Act of 1986 (PPL30/3445).

## Author contributions

EOA designed and performed experiments, analyzed data, contributed to the discussion, and wrote and revised the manuscript. CMW, YL, MTJVDB, SFM, AM, and LH performed experiments and analyzed data. IH contributed to the discussion and revised the manuscript. ASV provided the transgenic mice and revised the manuscript. AWP designed research and cowrote and revised the manuscript.

## Supplementary Material

Supplemental data

## Version 1 05/17/2018

Electronic publication
