# Supplementary material for: Aquaporin-1 regulates platelet procoagulant membrane dynamics and in vivo thrombosis
Source: JCI Insight. 2018 May 17;3(10):e99062. doi: 10.1172/jci.insight.99062 (PMC6012506; doi:10.1172/jci.insight.99062)
Supplement: Supplemental data [file jciinsight-3-99062-s101.pdf]

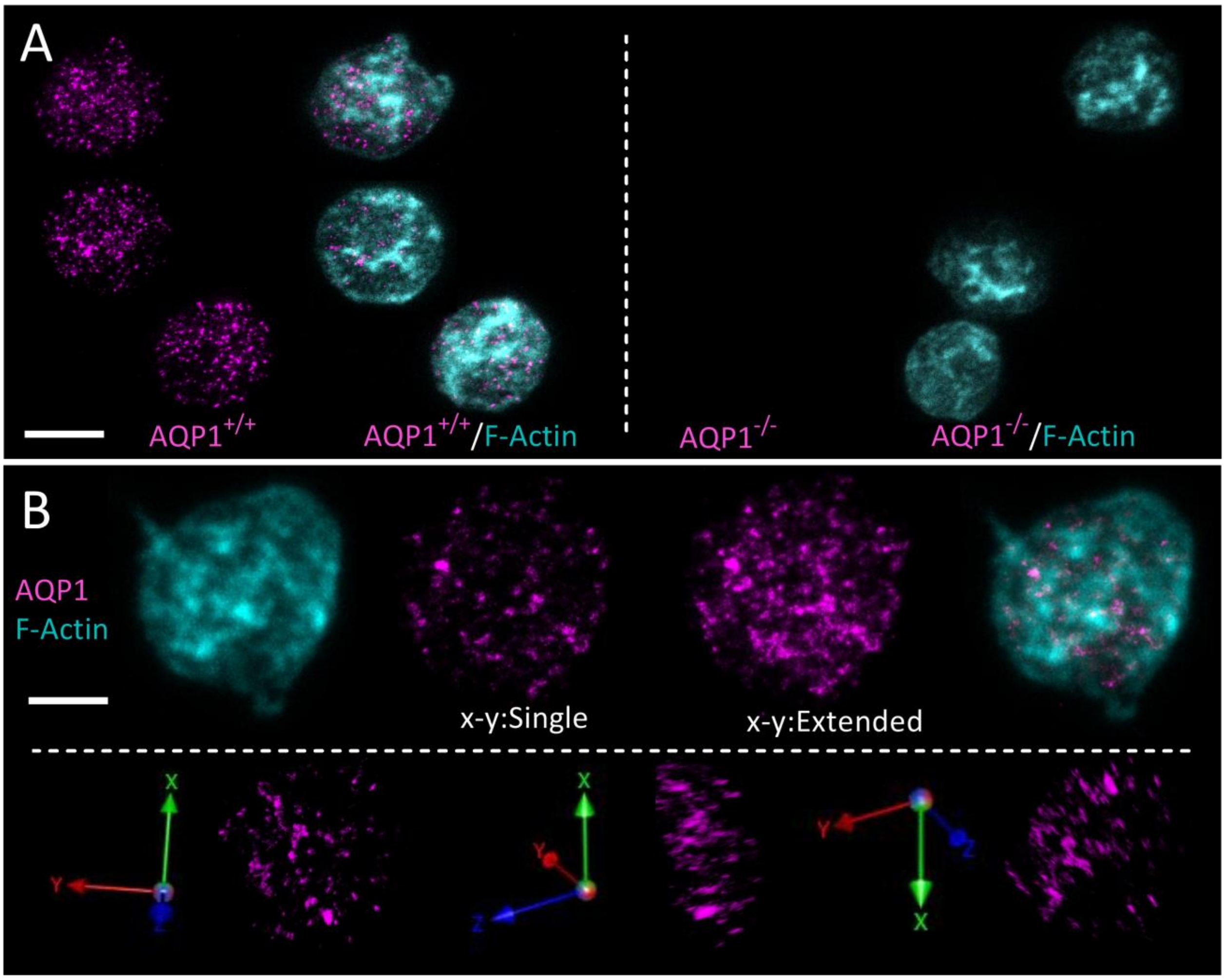

Figure S1: Expression of the water channel aquaporin-1 (AQP1) in mouse and human platelets is imaged by super-resolution microscopy. A: representative images of unstimulated wild-type and AQP1 knock-out mouse platelets. **B:** representative images of unstimulated human platelets. In A and B, platelets were probed for AQP1 (magenta) and F-actin (cyan blue) by immunocytochemistry and imaged by Stimulated Emission Depletion (STED) Microscopy. Lower panel B shows 3D rotated images of human platelets AQP1. Scale bar represents 2 µM in A and B. Data were from 3 independent experiments.

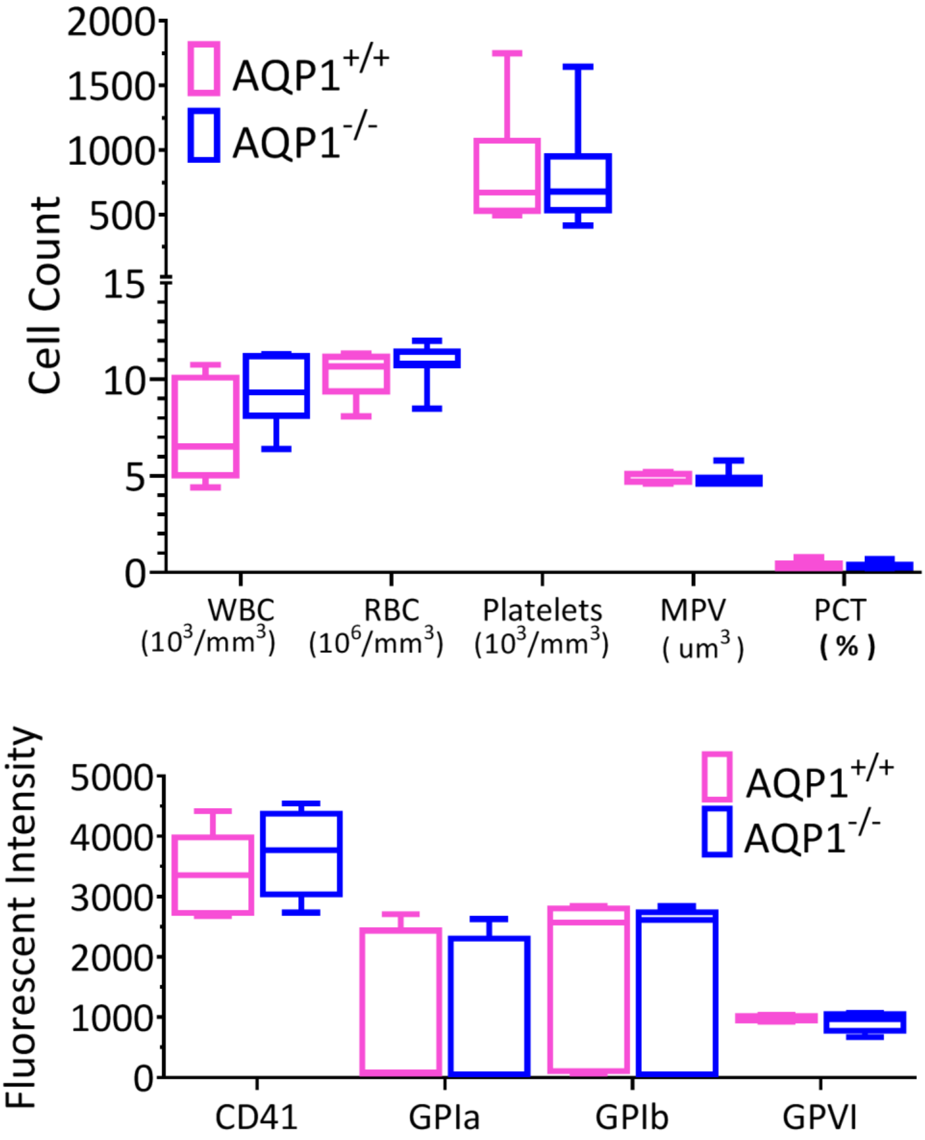

Figure S2: Haematological parameters and surface receptor expression in wild type (AQP1$^{+/+}$) and Aquaporin-1 null (AQP1$^{-/-}$) mice platelets. A: Whole blood from AQP1$^{+/+}$ and AQP1$^{-/-}$ mice was analysed by haematology cell counter (Pentra E60) for white blood cell count (WBC), red blood cell count (RBC), platelet count, mean platelet volume (MPV) and Platelet hematocrit (PCT) **B:** Using flow cytometry, the expression of key platelet surface glycoproteins in AQP1$^{+/+}$ and AQP1$^{-/-}$ mice was determined by an evaluation of FITC-conjugated monoclonal antibodies. Isotype antibody controls were used to account for nonspecific binding. Box and whisker plot showing minimum to maximum values, median, and interquartile range is shown; data were from platelets obtained from 7 mice.

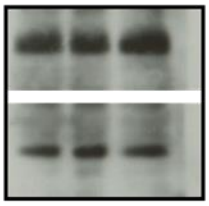

Figure S3: Human platelets express AQP 7

Washed human platelets (4 x 10^8/mL) were lysed and immunoblotted for AQP7. Tubulin was used as loading control. Each lane represents an independent sample.
